# “Soldiers of the System”: Maternity Care in Russia Between Bureaucratic Instructions and the Epidemiological Risks of COVID-19

**DOI:** 10.3389/fsoc.2021.611374

**Published:** 2021-02-10

**Authors:** Anna Ozhiganova

**Affiliations:** Center of Medical Anthropology, Institute of Anthropology and Ethnology (RAS), Moscow, Russia

**Keywords:** COVID-19, maternity care, childbirth, obstetricians, doulas, Partner birth, mistrust, bureaucratic logic

## Abstract

Preventive measures taken by the Russian maternity care system in response to the COVID-19 pandemic are very tough. Supporting persons (doulas and partners) are being completely excluded from the maternity hospitals. Pregnant women and newborns are distributed in different types of hospitals according to their epidemiological status (confirmed, suspected, contact, or “clear”). Severe infection control measures are introduced for women with confirmed or suspected COVID-19: separation from newborns and weeks of hospital quarantine. How do obstetricians and other perinatal specialists perceive these measures? What strategies do they choose and what new practices are being created? The study is based on interviews conducted between March and August 2020 with obstetricians-gynecologists, midwives, perinatal psychologistsdoulas, and women who gave birth during the pandemic and is focused on their subjective interpretations of COVID-related changes in maternal care. My data indicate that this pandemic with its high risks and uncertainties reveals multiple ethical and organizational conflicts among bureaucratic, managerial and professional logics in Russian health care in which mistrust has played an important role.

## Maternity Care in Russia: Bureaucratic Control and Institutional Mistrust

Maternity care reforms carried out during the post-Soviet period have ambiguous and contradictory consequences. On the one hand, reforms led to the commercialization of maternity care and the emergence of paid services and private maternity hospitals. Medical care is provided free of charge to all Russian citizens in accordance with the state health insurance program. However, women from a new category of demanding and informed consumers often pay for a “birth contract” in order to receive personalized care and more comfortable conditions in the hospital (Temkina 2017). The Rule of Informed Voluntary Consent allows women to refuse unwanted medical manipulations (Federal Law No. 323, 2011). The attendance of a birth partner is also guaranteed by the law: the child's father or other family members can accompany women in the birthing room (ibid). Doulas are also allowed to accompany women in some maternity hospitals, although their status remains uncertain. In general, maternity hospitals have become more open and more focused on the needs of women and newborns than two decades ago, at least in big cities: the practice of “soft” or “natural” childbirth is becoming more widespread, the “golden hour” after childbirth is respected, and breastfeeding is encouraged (Ozhiganova 2020).

On the other hand, from Soviet times to the present day, the logic of bureaucratic control continues to play a decisive role in the Russian healthcare system and has even increased in recent years (Litvina et al., 2020). The threat of prosecution against doctors has intensified, as evidenced by several high-profile trials of obstetricians-gynecologists and neonatologists. Russian doctors do not have the same expert power and autonomy as their counterparts in Western societies, medical professional organizations do not have much influence, and the economic and political interests of doctors are largely ignored (ibid.).

Homebirth is illegal; nevertheless it exists, at least in big cities, as an expression of mistrust of obstetric practice (Ozhiganova 2019). The number of out-of-hospital births is unknown because these statistics are not kept.

Confirming Fukuyama’s characterization of Russia as a “country of distrust” (Fukuyama, 1996), Russian citizens demonstrate an exceptionally high level of distrust in medicine. More than half of Russians (57%) do not consult a doctor in the case of illness, preferring self-medication; nearly a fifth of all citizens (19%) try to avoid doctors on principle (Health Mail.ru, 2019). Only 11% agree with the statement that a doctor is interested in their health (FOM 2019). The high vulnerability of doctors and the high risks of their work contribute to the fact that they themselves are not inclined to trust the system in which they work (Litvina et al., 2020).

During the coronavirus pandemic, Russian authorities have taken the infection control measures that are typical of authoritarian regimes: distortion of information, manipulation of statistics and outright disinformation; human rights violations—in particular, forced “self-” isolation; forced hospitalization of people with suspected COVID-19; and control of individuals via electronic passes containing barcodes through the Social Monitoring program, which tracks people’s locations and movements (Inozemtsev 2020). Additionally, institutional gaps in healthcare management and a lack of personal protective equipment (PPE) for doctors have resulted in the punishment of medical workers for complaints and in reprisals against independent medical organizations (Vasilieva 2020).

The fight against the pandemic in Russia is taking place in a situation of a new “legal void” or “counterfeiting of legality” produced by Putin’s government (Karaseva 2020: 294). Russia’s authorities are not using either of two versions of emergency regimes (“an emergency situation” and “the state of emergency”) provided by Russian law, but rather declared pre-emergency “high alerts,” and in amendments to these decrees introduced a “regime of self-isolation,” “distance work” and “quarantine”—all absent in the law. In the healthcare realm, this legal void has manifested in the mass diagnosis of “community-acquired pneumonia” instead of coronavirus infection (See, for example, the investigative journalism of Yapparova et al., 2020).

## Methods and Materials, Trust and Mistrust

In recent works, Mühlfried calls for a revision of the existing social science approach to the phenomenon of mistrust “as the flip side of trust, as an annoying absence, a societal failure, or an obstacle to be overcome” (Mühlfried, 2018:7). He suggests that trust and mistrust cannot be understood as opposites: those relationships that are often attributed to mistrust in fact are examples of the coexistence of trust and mistrust that emerge in situations of uncertainty. In the case of trust, people invest in the strengthening of their relations; in the case of mistrust, in the weakening of these relations and a translocation of trust into new trust networks. In order to define “mistrust” as an empirical phenomenon, we need to ask the questions: “How does mistrust work?” and whether or not mistrust itself may be shared and create bonds (ibid: 19). According to Mühlfried, mistrust is a reasonable reaction toward all kinds of revelations and may also be the first step toward critical political engagement.

The epidemic of COVID-19 in Russia triggered many latent conflicts in which mistrust played an important role. In this article, I ask, how has maternity care responded to the challenges of the COVID-19 epidemic? Given their general mistrust of “the system,” how are obstetricians reacting to the changed situation: new guidelines, anti-epidemic restrictions, and changed working conditions? What new relations and practices of trust and mistrust have emerged between perinatal professionals and women?

This article is based on interviews I conducted with 11 obstetrician-gynecologists, two midwives, two perinatal psychologists working in maternity hospitals, 6 homebirth midwives, 12 doulas, and 14 women who gave birth during the pandemic^1^. The first interviews were recorded in March, when the COVID-19 epidemic in Russia was just beginning; the last in August, when some preventive infection control measures had already been lifted. Thus it became possible to see what in the maternal health system changed initially and which changes lasted over time. Most of these recorded interviews were with perinatal specialists and women from Moscow and the Moscow region; seven were with representatives of other regions: Central, St. Petersburg, the Ural, and Siberia. I also followed the publications of an ob/gyn who blogged about his work at the Moscow COVID-19 maternity hospital on the Instagram social network (a very rare practice among Russian doctors), as well as the official pages of maternity hospitals on Facebook and Instagram.

My interviews with doctors and midwives included the following questions: How has your hospital's operating schedule changed? Has your obstetric practice changed? How do you assess the COVID-19 prevention measures in your hospital? My interviews with women included questions about whether the epidemic affected where, how and with whom the birth took place, and what factors were most influential. Since doulas usually know very well what is happening in the maternity hospitals of the city where they work, they have become valuable interlocutors; however, the focus of my research was on doctors and their perceptions of the pandemic situation. I had several points of entry into the field: I used old contacts with doctors and midwives, but also found new interlocutors through the Association of Professional Doulas and the Center for Traditional Midwifery, which conducts training courses for obstetricians-gynecologists and midwives. It should be noted that many doctors refused to be interviewed. For those who agreed, it was extremely important that the interview was not “official,” that is, they were guaranteed complete anonymity. I analyzed these interviews thematically.

In order to maintain anonymity, all personal names and names of maternity hospitals are not given. In interviews with doctors and midwives from the province, at their request, only the region is indicated, not the city, as this might make the data source potentially identifiable. Given the level of mistrust in the Russian healthcare system in general that my physician interlocutors expressed, readers may wonder why they were open enough with me as a researcher to answer my questions as frankly as they did (see below). Perhaps this openness was due not only to the anonymity I promised them, but also to my position, which I voiced before each interview: my goal is not to identify possible violations of the rules and protocols in their work, but to better understand how and in what conditions they have to work in this difficult situation of the coronavirus pandemic. It is also important to note that both in the late Soviet and post-Soviet traditions, there is a great distance between private conversation, in which people speak freely, and public speaking, in which people are generally very careful about what they say. My interlocutors perceived the interview as a private conversation.

## The Maternity Care Emergency Response to the Covid-19 Pandemic:Doctors’ Opinions

### COVID-19 Prevention Measures: Conversion of Maternity Hospitals, New Clinical Guidelines and Routing Plans

In the middle of March, the national Ministry of Health and regional Health Departments adopted a series of preventive measures in response to the COVID-19 pandemic. All maternity hospitals were divided into three groups: “clean,” “infectious,” and “buffer” (an intermediate zone, in which patients with an unconfirmed diagnosis are located) or respectively “green,” “red,” and “yellow” zones. The flows of pregnant women, women in labor, and newborns should be separated based on their COVID-19 tests, acute respiratory viral infection (ARVI) symptoms, and data on contacts with COVID-19, and directed to the appropriate hospitals according to the new routing plans. A quarantine regime was declared in all hospitals, meaning that visits to patients and partners at births were prohibited. Additional preventive measures were introduced for women with confirmed or suspected COVID-19: separation from newborns, prohibition of breastfeeding, and long-term quarantine in the hospital until negative test results are received (Guidelines 2020). Infants with neonatal disorders and COVID-19 suspected or positive should be sent to receive high-tech medical care in specialized hospitals, where special “Melzer boxes”—a completely isolated ward for infectious patients, with a gateway for staff—should be opened.

### Converting Maternity Hospitals to COVID-19 Hospitals

In the beginning of the epidemic, some maternity hospitals were converted to COVID-19 hospitals, where ob/gyns do not attend births, but work as general practitioners. The doctors explained that this solution was convenient, since in Russia maternity hospitals are usually detached buildings, with a “box” system, built as infectious disease hospitals where a strict sanitary and epidemiological regime is always observed. Due to the conversion of these maternity hospitals, the remaining “clean” hospitals received sharply increased patient flows. According to some reports, the number of patients in such hospitals has more than doubled: the same number of doctors and midwives began to take over 40 deliveries per day instead of the previous 20 (Interview 1b). Some maternity hospitals specializing in treating pregnant women with chronic diseases were also closed; as a result, pregnant women with heart or kidney problems could not receive all the necessary medical care (Interview 5).

One of the largest Moscow maternity hospitals, with 210 beds, was turned into a hospital for patients with COVID-19 on March 12. This news was reported by the media as a doctors’ initiative:

The staff of the maternity hospital referred to the Moscow Department of Health with a proposal to redesign their beds for an infectious disease hospital. Doctors explain it this way: “It is our professional duty to protect citizens.” (Protsenko 2020).

Dr. N., an ob/gyn of this maternity hospital, said that the decision about converting was made by this Department, and it could not be otherwise: such decisions are not made by the heads of hospitals, and still less by the staff:

What is the initiative? This is ridiculous. Of course, it is the Department's initiative. In our country, after all, everything is so—“at the numerous requests of the working people.” We all turned off instantly. Get up and go (Interview 1a).

As a result, Dr. N. and her colleagues worked for about three months as general practitioners with COVID-19 patients; the maternity hospital returned to its usual work at the end of July. Many of her colleagues were ill with COVID, and many of those ended up in intensive care. However, Dr. N. did not complain, noting that they were literally “bombarded with all sorts of benefits”: provided with PPE, paid allowances, brought good food and even offered rooms in 5-star hotels. However, it was very difficult for her not to do her job, and she doubts the correctness of such a decision: “We are deprived of our work, it is awful! It seems to me that this is just some kind of ineffective use of human resources” (Interview 1a). However, she believes that nothing could be done; they could only obey. Only two doctors from the large hospital staff left the service.

### Formal Cancellation of Partner Support and Informal Ways to Get Around it

Births with partners have become quite popular, especially in big cities: 30% in Moscow, and up to 70% in some maternity hospitals in Moscow and St. Petersburg. The cancellation of partnered births was painful for the women, who often searched for ways to get around it; some even decided to give birth at home. However, according to the homebirth midwives I interviewed, the pandemic did not significantly affect the number of out-of-hospital births: these were scheduled home births with a midwife.

Some maternity hospitals started to allow partners in July, but almost exclusively under contract (which actually meant that the couple had to pay for the presence of a partner) and, with rare exceptions, only fathers, not doulas. Most of the maternity hospitals, especially in the provinces, had not returned to this option by late August.

The prohibition of partnered birth has become one more manifestation of a “legal void,” since it has no legal basis. According to the Ministry of Health Guidelines, “partner birth should be prohibited in probable or confirmed cases of COVID-19 to reduce the risk of infection” (Guidelines 2020: 23), but in practice it was canceled for all.

Most doctors reacted very calmly to this prohibition, since they considered it to be a routine preventive measure. Dr. A., an ob/gyn at one of the Moscow maternity hospitals, believes that it is undoubtedly correct and an “absolutely ordinary quarantine measure” that is carried out regularly, every year, during the flu and ARVI season. She emphasizes that the restrictions affected only women, and for her, as a doctor, nothing has changed dramatically: “It’s just the work we do. These are the Ministry of Health’s Guidelines. We are obliged to obey” (Interview 3).

Dr. E., an ob/gyn of the St. Petersburg maternity hospital, where births with partners accounted for 50% of all births, also unequivocally supports their cancellation: “This is an adequate measure: the fewer contacts, the less chance of infection.” As confirmation, she told a story about an event that occurred in her maternity hospital during the swine flu epidemic in 2009: a husband visited his wife and as a result she fell ill and died. E. is convinced that women understand this prohibition: “I have not seen anyone resent it, because everyone understands that this is how the whole country lives, and no one is to blame. As a matter of fact, there is no one to make claims to” (Interview 4).

However, other doctors admit that women do not agree with the prohibition of partnered births and express their protest. Dr. V., the head of the maternity hospital in the Ural region, said that she is constantly faced with the demands of women to allow accompanying partners:

Just recently there was a woman who was extremely negative, and I told her: contact the Ministry of Health and Rospotrebnadzor (Federal Service for Surveillance on Consumer Rights Protection and Human Wellbeing] since this is not a requirement of a maternity hospital, this is a requirement of these bodies. After that, her husband called me and said: “Yes, we turned [to them], and we were told, please, at the discretion of the maternity hospital, they can allow partners (Interview 6).

However, Dr. V. did not allow him to attend his wife’s labor or birth; in her opinion, the officials were only trying to shift the responsibility to her: “These people (officials of Rosotrebnadzor] behaved unscrupulously, because if something happens, some outbreak in the hospital, then the head will answer—that is, I will answer” (Interview 6).

Partnered birth has turned into a rare and accordingly valuable service, and very quickly became the subject of all sorts of informal agreements and informal payments. Some maternity hospitals in the Moscow and St. Petersburg regions unofficially allowed partners to accompany women. Despite the order of the Health Department, a private Moscow maternity hospital continued the practice of partnered deliveries (in some cases, for one partner, and in some cases, for two) for the entire period of the epidemic in Russia. The doctor from this hospital confesses that everything remains as before but “unofficially” (Interview 2). At the same time, the contract price sharply increased, and became the reason for the joke, “In order for the coronavirus to become safe, you need to pay 350 thousand rubles; if the contract is 200 thousand or 150 thousand, then the virus is still very dangerous!” (Interview 8).

Some women decided to use a service provided by some maternity hospitals: accompaniment by a perinatal psychologist, a hospital staff member. Perinatal psychologists confirmed that the number of requests for their services increased during the epidemic. However, it turned out that not all women are satisfied with this option, since they fear that such a partner is not acting in their interests: “She will play along with the doctors, to persuade me to do something, maybe not what is best for me, but what is more convenient for a doctor, because then she will continue to work with him, and I will leave” (Interview 8). Instead, women tend to trust doulas because they are from outside the healthcare system. In such cases, women prefer online doula support over the support of a hospital psychologist.

The doulas protested the cancellation of partner births: they prepared a petition and called on women to fight for their rights. In the middle of March 2020, a doula and a lawyer, M., published on social networks a proposal to write requests to Rospotrebnadzor demanding an explanation of this measure. M. considers it illegal: since a state of emergency was not declared, the guarantees of citizens' rights established by law cannot be canceled. However, no doula initiatives received noticeable support. In some cases, partners were allowed when, on the advice of this doula, they demanded a written refusal with reference to the law. Such informal negotiations turned out to be very limited but were the only way to solve the problem of achieving partnered birth.

### Maternity Hospitals for Women with COVID-19: Epidemic Expediency or Additional Risks to the Health of Women and Newborns?

Admission into a COVID-19 maternity hospital means that very harsh measures will be applied to a woman and her newborn: separation immediately after birth, and very often increased medicalization and even use of drugs that are prohibited for pregnant and lactating women: Kaletra (which is used in HIV treatment), Azithromycin, and other antibiotics. My interlocutors noted an increase in perinatal losses due to spontaneous abortions and intrauterine fetal deaths (Interview seven; Charitable Foundation "Light in Hands").

According to my interlocutors, obstetric practice has changed dramatically since the advent of COVID-19 and the number of surgical interventions in such hospitals has increased. A midwife from the Siberian region said that in her maternity hospital, designated for women with COVID-19, the number of cesarean births increased from 25% to about 60–70% because many doctors do not even give women the opportunity to enter into labor, but immediately send them to surgery (Interview 7). It should be noted that this even happened in a maternity hospital known for its support of natural birth; for example, this hospital previously allowed vaginal births after cesareans (VBACs) even if the mother had experienced two previous cesarean deliveries.

The first maternity hospital, which, in accordance with the recommendations of the Ministry of Health, was designated "to receive pregnant women with ARVI, community-acquired pneumonia and patients who are quarantined due to contact with coronavirus infection" (Guidelines 2020) began working in Moscow on March 31. It is a large 170-bed maternity hospital with more than 7,000 births per year. In April, it received a fairly large number of patients: three to four per day, some with severe symptoms of COVID-19. However, in July, an ob/gyn of this hospital wrote on his Instagram blog that they had very few patients at that time: “Honestly, there is practically nobody to treat. There are only 15 patients in the huge maternity hospital building!” (Doctor_yakunin Instagram post 3).

A 60-bed maternity hospital in a large Siberian city was assigned to work with women with COVID-19 on April 27. It accepts women from all over the city with suspected coronavirus infection, and according to midwife T., all the time there were on average about 10–12 women in all three departments. Women are tested in the admission department and placed in the “yellow” buffer zone, then based on the test results, after 3–4 days, they are transferred to the “green” or “red” zone.

Thus, those maternity hospitals that retain their status as infectious disease hospitals are only partially filled. My interlocutors say that very often women are brought to them without symptoms and with an unconfirmed diagnosis:

An ambulance brought a woman with a screaming seven-day-old baby. The woman is worried about pain in the seam (scar) after cesarean section, which was made in an ordinary “clean” maternity hospital a week ago. After discharge, she went to her mother-in-law to pick up the older child. It turns out that this grandmother has IgG antibodies to coronavirus (he presence of these antibodies indicates the presence of an immune response, i.e. disease resistance) (Doctor_yakunin Instagram post 3).

Thus, we can see that an ambulance, by order of the Department of Health, brought women to this COVID hospital without sufficient reason (allegedly this particular woman was in contact with an infected person), just so that it would not be empty.

A similar situation has developed in the Siberian maternity hospital. By order of the regional Department of Health, it should accept women with a temperature above 37 Celsius (98.6 Fahrenheit), or with the signs of ARVI (acute respiratory viral infection), and with an obstetric pathology. However, many doctors accept pregnant women with only mild signs of a cold (Interview 7). Doctors often assess the Department's order to send women with a runny nose to an infectious maternity hospital as “absolutely absurd” and advise their patients to drip a vasoconstrictor before admission (doctor_yakunin Instagram post 1).

Doctors also understand that during childbirth, body temperature can rise due to a psycho-emotional factor, or simply because of the summer heat, or because of kidney problems, but often doctors in an ambulance do not take this into account and take the patients straight to the infectious disease hospital (Interview 7).

A woman who finds herself in a buffer (“yellow”) maternity hospital (or department) also must expect a rapid cutting of the umbilical cord and separation from the child. On the official Facebook page of one of these maternity hospitals, women are told that they will have to stay in the hospital for at least two weeks during the incubation period of coronavirus infection: “We will not dismiss you if after a couple of days you feel great, because you can be a carrier of a mild illness and pass it on to others” [36roddom (maternity hospital 36) Facebook post].

Women who seek medical help due to symptoms of ARVI at any stage of pregnancy are at risk of forced admission to such a hospital. It is not surprising that some women, when they feel unwell, self-medicate and think only about hiding their symptoms from doctors. One of my interlocutors said that she and her family most likely had COVID-19 in April: for two weeks she had a fever, severe weakness and cough. She treated herself with homeopathic remedies, did not go to doctors, and in August, a month ahead of schedule, gave birth to a healthy child (Interview 9).

Doctors are ambivalent about the separation of mothers from newborns. Some believe that this measure is rational, because they believe that presently there is “too little data” on the transmission of the disease from mother to child and “it is better to be safe just in case.” (Interview 3, 4). Others admit that this measure is too harsh (Interview 5), that they do not consider it reasonable either from an epidemiological or psychological point of view: “Mothers are being treated here in the hospital, either they have coronavirus, or it is an error in the analysis. We take the next analysis after 10 days, and they lie all this time, gargle, drip their nose, and cry for their babies” (Interview 7).

When asked how it is possible to obtain women’s consent for such treatment, T. says that doctors always have the opportunity to intimidate, to say that it is dangerous for a child to be with his mother, that he will get sick and may die. One of my interlocutors, a woman ob/gyn who is herself an expecting mother, confirms that she not only supports the separation of mother and child, but she is ready, if necessary, to be separated from her baby immediately after the birth: “I would prefer that my child had less opportunity to get infected from me. I would rather refuse to stay together if only I understood that my child is being cared for, that he is fed and safe” (Interview 4).

The Russian Association of Natural Feeding Consultants (ANFC) opposed the practice of separating mother and child in maternity hospitals. In an open letter to the Minister of Health dated July 31, 2020, members of the Association stated that "the real risks to the health of mothers and newborns due to lack of contact and the prohibition of breastfeeding are higher than the potential risk of COVID-19 infection" and demanded that hospitals not separate COVID-positive mothers from their newborns if the mother's condition is not serious, and to ensure the right of newborns to breastfeeding in all cases, observing the antiseptic methods proposed by WHO recommendations (wearing a mask, washing hands and disinfecting surfaces) (ANFC 2020). However, the doctors did not support the initiative, and the Ministry responded with a formal refusal.

During the epidemic, many doctors found themselves in a difficult situation, especially in the provinces. According to unofficial data, the death rate of doctors from COVID-19 in Russia is much higher than in other countries (Medvestnik 2020). My interlocutors from provincial maternity hospitals confirm that disposable PPE is in short supply, so they wash and dry it in the hospital. Many doctors, midwives and nurses working with COVID-19 patients have not received the incentive payments promised to them by Presidential Decree on May 6. Midwife T. says that she was ill with COVID-19 in May but has not received any insurance payments. She said that some her colleagues are already planning to quit after the epidemic: “It is simply impossible to work; all the problems came out that we did not pay attention to before, just because we were very busy with a large flow of patients” (Interview 7). She admits that the head of the hospital always behaved very rudely with the staff and did not seek to provide the hospital with everything necessary (in particular, no needed repairs were made for a long time).

Doctors may evaluate the introduced preventive measures in different ways, but if they consider some of them not useful or even harmful and absurd, they do not declare their disagreement publicly, but simply obey bureaucratic requirements and protocols. Physicians can warn their patients and advise them how to get around restrictive measures as a part of private relations of trust. They can express their disagreement by quitting their job, but in general they cannot affect the functioning of the system.

## Patients and Healthcare Practitioners: Practices of Separation, Prohibition, and Mistrust

This research was conducted during the “first wave” of the coronavirus pandemic (from March to August 2020) and does not cover the changes that have occurred later. The differences between central cities and the periphery and diversity in maternal care facilities that exist in a country as large and heterogeneous as Russia cannot be captured in such “quick” study. As a result, the picture turns out to be rather mosaic, however, taking into account these limitations, some preliminary conclusions can be drawn.

As demonstrated in the articles in this Special Issue, the responses of various national healthcare systems to the challenges of the COVID-19 pandemic often have a great deal in common, such as prohibiting or severely limiting visitors, doulas, birth partners, and post-birth mother-newborn contact and breastfeeding. Yet some of the measures taken in Russia are very different from those in other countries, such as the division of maternity hospitals into red, green, and yellow zones and the enforced long hospital quarantines for women and newborns “just in case.” Public discussions about the risks of these prohibitions have been conducted in many countries, and in some cases, for example, in New York, they were canceled due to public protest, largely from women, midwives, and doulas (Davis-Floyd, Gutschow, and Schwartz, 2020: 7). Yet in Russia, a discussion inspired by doulas in the electronic social networks passed almost unnoticed, as the discontent of women and doulas and their proposals for humanistic improvements were not supported by the medical community and health officials.

Unlike American women who are afraid of hospitals because of the possibility of contagion, (ibid: 8), Russian women fear COVID-19 much less than the restrictive measures introduced in maternity hospitals. A sad joke appeared: "In Russia, the coronavirus is not as terrible as the fight against it." Women are afraid to go to the hospital without a partner, fearing unreasonable medical interventions, and are even more afraid of the infectious disease maternity hospitals, where they will be separated from their babies immediately after birth and for the next weeks.

The main strategy of many pregnant women in the pandemic situation is the mobilization of all resources “to insure” against possible risks: they search for reliable information about doctors and maternity hospitals; make informal agreements with doctors; commit to expensive birth contracts; and generate agreements with doulas for remote support (via video or audio communication). Thus, the pandemic situation contributes to the increase in informal relations and informal payments in maternity hospitals, and, accordingly, to the increase in inequality among different social classes, as well as between the big cities of Moscow and St. Petersburg and the provinces.

The Guidelines of the Ministry of Health and the orders of regional departments, declared to be aimed specifically at “minimizing the risks” of the spread of coronavirus infection, contradict evidence-based medicine data and international recommendations, and some of them, such as separation of mothers from newborns and prolonged hospital quarantine, cannot be considered rational medical ethics and patient's rights are viewed as irrelevant and negligible, and the principles of separation and prohibition are authoritative. The principle of prohibition as applied in Russia justifies “the system’s” prohibitions as described above (see Benaglia, this issue). And according to Davis-Floyd (2003, 2018), the technocratic model of obstetrics is based on the principle of separation, in which mind is separated from body, the practitioner is separate from the patient—as in not emotionally connected to her--and, among other forms of separation, the mother is separated from both her support people and her baby. Under this ideology, it is easy to justify such separation without remorse. In contrast, the humanistic model as defined by Davis-Floyd (ibid.) is based on the principle of connection: connection of mind and body, of practitioner and patient, of the mother to her support persons, and of mother and baby. Pre-COVID, this principle of connection used to characterize maternity care in some of the more progressive Russian hospitals.

Why did the Russian system of maternity care react so harshly, canceling many progressive innovations of recent years, rejecting WHO recommendations and evidence-based medical data? My interlocutors from the older generation of doctors believe that this reaction is caused by "historical memory": in a situation of epidemic danger, health officials immediately reverted back to the old Soviet practice based on the principles of prohibition and separation, the dominance of bureaucratic logic, paternalism, and neglect of patient rights, when maternity hospitals were completely closed institutions with strict and prohibitive rules, separation of mothers and newborns, and severe sanitary and infection control measures. One obstetrician commented:

I still remember the old obstetrics. You are at war all the time. The maternity hospital is a field of military operations. Therefore, there were such strict midwives and nannies, because in fact, neither the woman nor the child was perceived as (a subject] of care. The emotional background was not taken into account. It was a very difficult psychological load, and on the staff too, because they were something like cogs of this machine (Interview 5). As my interviews show, doctors and midwives may disagree with these drastic changes, express their opposition to bureaucratic directives, and empathize with women, but they cannot state this opposition publicly. At the same time, it is clear that medical professionals are increasingly worried about their professional autonomy. In an emergency regime, which was not formally declared, the dependence of doctors on bureaucracy at various levels—from the head of the hospital to the Ministry of Health—became even more visible than in ordinary times. Russian doctors as “soldiers of the system” are obliged to follow the orders of health officials, and their professional position is regarded only as a private opinion. They cannot be sure that they will receive the necessary protection from infection and monetary compensation, nor do they have any leverage over the hospital administration and health officials. The pandemic situation reveals the fact that physicians themselves do not trust the institutions in which they work, as shown by the results of a study conducted by a group of sociologists in St. Petersburg hospitals (Borozdina and Novkunskaya, 2020). Doctors, just like patients, do not trust official information, which leads to criticism of the authorities' actions to combat the epidemic:

To be honest, I still don't really understand what’s going on. I am still in some incomprehensible state from all this, whether this is a great lie, or is it a great infection? (Interview 1b).

Today I have the opinion that we are somehow very systematically prepared for the fact that the coronavirus will densely enter our lives, and we will fight with it for many, many years. They want to intimidate us so that we can endlessly fight the coronavirus (doctor_yakunin Instagram post 2).

These doctors, who themselves work in the COVID hospitals, do not deny the existence of the virus; their mistrust is a variant of Covidian dissidence—a term widely used in Russian discourse, both in media and in electronic social networks) --which should be viewed as a specific way to express mistrust toward the authorities. Such doctors may indeed be “soldiers of the system,” forced labor within it and to obey its rules just as military soldiers must, but that does not mean uncritical acceptance of the system as it is nor of its rules. A new “legal void” produced by the government, lack of transparency in the actions of the authorities, and mistrust of official information about the real situation with this pandemic become reasons for reluctance and dissidence—for hesitation tinged with mistrust—to adhere to the measures of the healthcare system (Somparé and Somparé 2018: 130). Thus I argue that the pandemic as a situation with high risks and uncertainty reveals and highlights multiple latent conflicts in which mistrust has long played an important role in the Russian context. This dense tangle of problems could be untangled if both doctors and women would refuse the usual strategy of informally solving their particular problems and transition to a systematic problem-solving strategy that would involve public speaking, strengthening professional, patient, and women's organizations, and creating new practices of solidarity and trust between practitioners and patients. In such ways, doctors, with the help of women activists, could transform themselves into system changers rather than system “soldiers.”

## List of Interviewees

1. Interview 1a. N., obstetrician-gynecologist, state maternity hospital. Moscow, March 24.
2. Interview 1b. N., obstetrician-gynecologist, state maternity hospital. Moscow, August 5.
3. Interview 2. V., obstetrician-gynecologist, private maternity hospital. Moscow, March 26.
4. Interview 3. A, obstetrician-gynecologist, state maternity hospital. Moscow, March 30.
5. Interview 4. E., obstetrician-gynecologist, state maternity hospital. St. Petersburg, July 10.
6. Interview 5. O., obstetrician-gynecologist, medical center. Moscow, July 13.
7. Interview 6. C., obstetrician-gynecologist, head of the state maternity hospital. Ural region, August 9.
8. Interview 7. T., midwife, state maternity hospital. Siberian region, August 17.
9. Interview 8a. M., doula. Moscow, Marth 20.
10. Interview 8b. M., doula. Moscow, May 8.
11. Interview 9. K., childbirth during the COVID-19 pandemic. St. Petersburg region. July 11, August 14.

## Internet Resources

1. 36 roddom [maternity hospital 36]. Coronavirus and pregnancy. Facebook, April 1. https://www.facebook.com/36roddom/posts/2909028449184940/.
2. doctor_yakunin. How to give birth to a healthy baby now? Instagram post 1, April 20. https://www.instagram.com/p/B_M5KsUAgyJ/.
3. doctor_yakunin. Instagram post 2, July 21. https://www.instagram.com/p/CC57ul5gNhh/.
4. doctor_yakunin. Instagram post 3, July 24. https://www.instagram.com/p/CDA3BpJAAqX/.

## Data Availability

The datasets presented in this article are not readily available because this article is based on interviews I conducted with 11 obstetrician-gynecologists, two midwives, two perinatal psychologists working in maternity hospitals, 6 homebirth midwives, 12 doulas, and 14 women who gave birth during the pandemic. In order to maintain anonymity, all personal names and names of maternity hospitals are not given. All materials are kept in my personal archive. Requests to access the datasets should be directed to Anna Ozhiganova, anna-ozhiganova@yandex.ru

## Footnotes

1 Ethical approval and written informed consent for participation were not required for the study of human participants in accordance with the legislation of the Russian Federation and institutional requirements. Verbal informed consent for participation in the study and for publication of the results was obtained from all participants.
